# Severe Aplastic Anemia as First Manifestation of Classical Hodgkin Lymphoma

**DOI:** 10.1155/2021/8876249

**Published:** 2021-02-09

**Authors:** Cláudia L. Pedrosa, Patrícia Rosinha, Patrícia Seabra, Gisela Ferreira, Cláudia Rosado, Luísa Regadas, Cláudia Casais, Jorge Coutinho

**Affiliations:** ^1^Department of Clinical Hematology, Centro Hospitalar Universitário Do Porto, Porto, Portugal; ^2^Department of Internal Medicine, Centro Hospitalar Do Baixo Vouga, Aveiro, Portugal; ^3^Department of Clinical Hematology, Centro Hospitalar Do Baixo Vouga, Aveiro, Portugal

## Abstract

Autoimmune cytopenia, a known paraneoplastic complication of lymphoid neoplasms, may occur before, concurrently, at relapse, or even years after completion of lymphoma treatment. In the case of Hodgkin lymphoma (HL), it is thought that immune dysregulation, typical of this neoplasm, may be involved in the genesis of these manifestations. We report a 57-year-old male presenting with stage IIIA, International Prognostic Score (IPS) 4, nodular sclerosis HL, and severe AA (SAA) confirmed on the histologic exam of the bone marrow that showed severe marrow hypoplasia due to a decrease in the elements of the three cell linages with left shift of the myeloid maturation. Immunosuppression with steroids and cyclosporine A was started. Eltrombopag and G-CSF were also added. In spite of prompt initiation of immunosuppressive therapy, the patient presented an unfavorable outcome with progressive pancytopenia and severe acute cerebral hemorrhagic event. The patient died 59 days after admission. Although autoimmune disorders are described in HL, its concomitant diagnosis is extremely rare. Our case shows a rare instance of SAA as the first manifestation of HL.

## 1. Introduction

Hodgkin lymphoma (HL) accounts for approximately 10% of all malignant lymphomas, commonly affecting young adults. It usually presents with localized disease involving cervical, supraclavicular, and mediastinal regions. The classical form represents about 95% of the HL, nodular sclerosis being the most frequent subtype [1].

Autoimmune cytopenias are frequent paraneoplastic complications of non-Hodgkin lymphomas, but more rarely seen in HL. These may occur before, concurrently, at relapse, or even years after completion of lymphoma treatment [2].

The pathophysiology of this phenomenon is not completely understood. However, in HL, it is thought that immune deregulation, typical of this neoplasm, may be involved in the genesis of these manifestations.

Aplastic anemia (AA) is a hematopoietic stem cell disorder defined by reduced bone marrow cellularity and impaired peripheral blood cells production [3, 4]. It is classified according to the marrow cellularity and repercussion on the peripheral blood. Severe aplastic anemia (SAA) comprises bone marrow cellularity less than 25% and depression of at least two of the three hematopoietic lineages (absolute neutrophil count (ANC) <0.5 × 10^9^/L; platelets <20 × 10^9^/L; reticulocytes <60 × 10^9^/L). SAA designates patients who fulfill SAA criteria but present with an ANC <0.2 × 10^9^/L [5].

The underlying cause of AA seems to be an autoimmune process mediated by *T* cytotoxic cells that suppress hematopoiesis. Coincidentally, these cytotoxic T lymphocytes are abundantly present in HL, playing an important role in the pathogenesis of the disease and in tumor cells' survival and proliferation. This fact raises the possibility of a causal relationship between the two diseases.

Here, we report a rare case of a patient presenting SAA as the first manifestation of nodular sclerosis classical HL.

## 2. Case Report

A 57-year-old male, with no comorbidities, Eastern Cooperative Oncology Group (ECOG)-Performance Status 1, presented with asthenia, lower gastrointestinal and mucocutaneous bleeding, and right axillar adenomegaly, two months before admission.

Blood workup showed pancytopenia and reticulocytopenia with no morphologic atypia on the peripheral blood smear. Erythrocyte sedimentation rate was elevated. All tumor markers, including *β*2-microglobulin and LDH, were negative. The patient had neither renal nor hepatic impairment. There was neither hemolysis pattern nor vitamin deficiencies. Microbiologic and autoimmune screening was also negative (Table 1).

A PET-CT scan was performed and documented hypermetabolic activity on supra- and infradiaphragmatic adenopathies and spleen. No bulky mass was identified.

**Figure 1:**
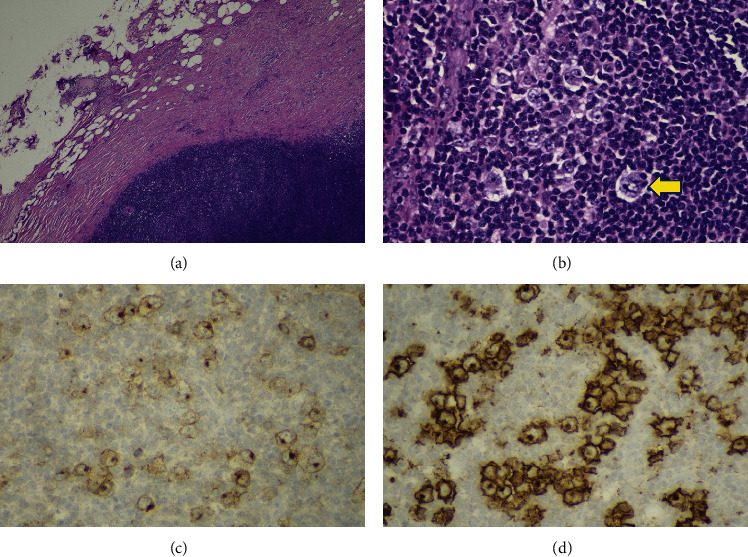
Histology and immunochemistry of axillar lymph node. (a) Lymph node with enlarged capsule and disruptive morphologic structure (hematoxylin-eosin staining). (b) Reed–Sternberg cells (arrow) (hematoxylin-eosin staining). (c) Redd–Sternberg cells showing low affinity to CD30 antibody. (d) Redd–Sternberg cells showing high affinity to CD15 antibody.

The excisional biopsy of the axillar lymph node confirmed the diagnosis of nodular sclerosis classical HL (Figures 1(a)–1(d)).

**Figure 2:**
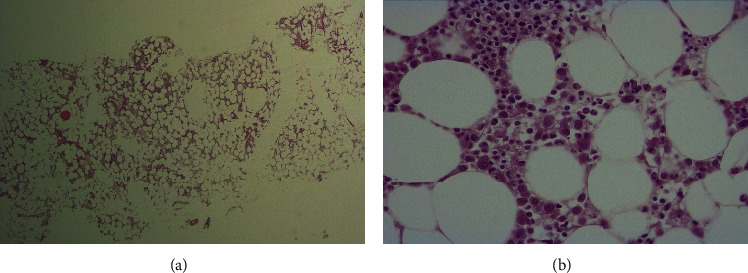
Histology of bone marrow (hematoxylin-eosin staining). (a) Severe marrow hypoplasia (cellularity between 10% and 30%). (b) Histologic exam of bone marrow showing reduced presence of elements of the three cell lineages with left shift of the myeloid maturation. No blastic, dysplastic, or lymphoma cells were identified.

A bone marrow biopsy was performed. The histologic exam revealed severe marrow hypoplasia due to a decrease in the elements of the three cell linages with left shift of the myeloid maturation. No blastic, dysplastic, or lymphoma cells were identified (Figures 2(a)–2(b)). Karyotype was normal (46, XY).

Next generation sequencing gene panel excluded mutations suggestive of inherited bone marrow failure syndrome or hypoplastic myelodysplastic syndrome/acute myeloid leukemia (Table 1). Immunophenotyping of the peripheral blood excluded paroxysmal nocturnal hemoglobinuria clone.

The concomitant diagnosis of advanced stage (Ann-Arbor IIIA) nodular sclerosis HL, IPS 4, and SAA was made.

Considering the myelotoxicity of standard HL's chemotherapy regimens, it was decided to start immunosuppressive therapy for SAA and postpone the introduction of chemotherapy. Prednisolone (1 mg/kg/day) was initiated. After one month on corticosteroids with no response, cyclosporine A was added. On day 15 of double immunosuppressive therapy, an agonist of thrombopoetin (eltrombopag 150 mg per day) was introduced.

After thirty days on immunosuppression, no improvement was documented on the blood counts (Hb <7 g/dL; neutrophils <1 × 10^9^/L; platelets <10 × 10^9^/L). G-CSF was started. During hospitalization, the patient was dependent on red blood cells and platelets transfusion.

Despite all the adopted measures, the patient's clinical status worsened and he was admitted to intensive care unit (ICU) for septic shock with multiorgan failure. Broad spectrum antibiotics and ventilatory and cardiovascular support were initiated. A multisensitive *Escherichia coli* was isolated on the peripheral blood cultures.

On the third day at the ICU, neurologic deterioration was noticed. A brain CT scan was performed showing “de novo” acute subdural hematoma and hydrocephaly.

Due to refractoriness to the immunosuppressive therapy and progressive deterioration of performance status of the patient in the context of septic shock and severe acute cerebral hemorrhagic event with hydrocephaly, it was decided to withdraw measures and begin palliative care.

The patient died 59 days after admission.

## 3. Discussion

Classical HL is characterized by the presence of B cells derived from germinal center, called Hodgkin or Reed–Sternberg cells (HRS). Although these cells derived from B lymphocytes, they do not express the majority of germinal center makers, do not produce immunoglobulins or undergo somatic hypermutation, under normal circumstances, a cell like this would be destined to apoptosis. However, in HL, the environment surrounding the HRS is the main element for tumor cell survival [6].

Autoimmune disorders are known complications of HL. Data suggest that HL's patients present a higher incidence of AA compared to the general population [7]. This may possibly be justified by the dysregulated immune response characterizing these neoplasms. In HL, an inflammatory background, constituted by *T* and B cells, macrophages, histiocytes, eosinophils, neutrophils, and plasma cells secreting cytokines, plays an important role in the survival of the HRS. In their turn, these cells also produce multiple cytokines and chemokines, such as tumor necrosis factor alpha (TNF-*α*), interleukin-4 (IL-4), IL-5, IL-6, chemokine ligand-5 (CCL-5), and fibroblast growth factor (FGF), which perpetuate this inflammatory activity [1].

The constitutive activation of the nuclear factor-kappa B and the Janus kinase-signal (JAK-STAT) signaling results in the upregulation of programmed death 1 ligand (PD1-ligand) and JAK2. This allows the interaction between the PD1-ligand on the surface of the HRS and PD1 on the T cell, downregulating the latest and compromising the function of cytotoxic T cells. Not only these cells but also *T* helper lymphocytes play a role in promoting tumor cell survival.

This immune dysregulation may represent a risk factor for AA [7].

Considering AA as a paraneoplastic manifestation of HL, the reasonable was to treat the underlying cause: the lymphoma. However, current chemotherapy regimens for HL are extremely myelotoxic and would not be feasible in a scenario of severe pancytopenia.

In recent studies, cases in which AA and HL arise simultaneously, the outcome of AA prevails over that of the lymphoma in the majority of the cases. The initiation of immunosuppressive treatment for AA plays a role in avoiding permanent and irreversible damage in the myelopoiesis [8]. The major challenges of these treatment regimens are severe complications, such as febrile neutropenia and infections by atypical or opportunistic agents, due to deep prolonged pancytopenia and extreme state of immunosuppression. In this case, we decided to start a mild-moderate immunosuppressive therapy in order to improve peripheral blood counts, allowing the patient to start chemotherapy. However, that was not possible due to sudden unfavorable progression of the patient's clinical situation. Nowadays, the advent of novel agents allows treating these patients with fewer side effects, mainly concerning myelotoxicity. Unfortunately, at the time of diagnosis, neither brentuximab nor checkpoint inhibitors were approved in Portugal.

To conclude, we consider the early recognition of the association between these two entities extremely important, since it allows a more accurate diagnosis and thus prompt initiation of effective immunosuppressive therapy. Despite being a malignant disease, HL shows high rates of complete remission and overall survival. On the other hand, AA is a benign disease with high rates of relapse/refractoriness to treatment and complications, certainly having an evident negative impact on the outcome of these patients.

## Figures and Tables

**Table 1:** Patient's initial blood workup.

Laboratorial hematology	
*Hemogram*	
Hemoglobin	5.8 g/dL
Leucocytes	2.8 × 10^9^/L
Neutrophils	1.36 × 10^9^/L
Platelets	<10 × 10^9^/L
Reticulocytes	26 × 10^9^/L
Erythrocyte sedimentation rate	92 mm/h

*Biochemical profile*	
Creatinine	65.43 *μ*mol/L
Alanine/aspartate aminotransferase	28/32 U/L
Total bilirubin	15.56 *μ*mol/L
Triglycerides	1.12 mmol/L
Ferritin	553 *μ*g/L
LDH	221 U/L
*β*2-microglobulin	1.98 mg/L

*Immunologic screening*	
Antinuclear antibodies (ANA) (immunofluorescence) Antineutrophil cytoplasmic antibodies (c-ANCA) (immunofluorescence) Antineutrophil cytoplasmic antibodies (p-ANCA) (immunofluorescence)	<1/160 (1/160) <1/20 (<1/10) <1/20 (<1/10)

*Serological/microbiological screening*	
Interferon gamma release assay (IGRA) for *Mycobacterium tuberculosis* infection (*in vitro* stimulation)	<0.01 UI/mL
Human hepatitis virus B (HBs antigen)	<0.10 (<1.00)
Human hepatitis virus B (HBs antibody)	0.16 (<1.40)
Human hepatitis virus B (HBs antibody)	13.78 m UI/mL (<8.00)
Human Hepatitis virus C antibody	0.21 (<1.00)
Human immunodeficiency virus	0.10 (<0.90)
Epstein-Barr virus, viral capsid antigen (VCA) IgM antibody Epstein-Barr virus, viral capsid antigen (VCA) IgG antibody	0.40 RU/mL (<0.80) 97.10 RU/mL (<16.00)
Cytomegalovirus, IgM antibody Cytomegalovirus, IgG antibody	0.15 UA/mL (<0.90) 11.80 UA/mL (<0.90)
Parvovirus B19, IgM antibody (immunoassay) Parvovirus B19, IgG antibody (immunoassay)	Nonreactive 3 UI/mL
Venereal Disease Research Laboratory (VDRL) test *Treponema pallidum* haemagglutination assay (TPHA)	Nonreactive Negative
Rubella virus, IgM antibody Rubella virus, IgG antibody	0.00 UI/mL (<0.80) 16.50 UI/mL (<5.00)
*Toxoplasma gondii*, IgM antibody *Toxoplasma gondii*, IgG antibody	0.20 UI/mL (<0.90) 5.20 UI/mL (<6.40)
*Borrelia burgdorferi* screening antibodies (enzyme-fluorescent assay)	Nonreactive
*Bartonella henselae*, IgM antibody (indirect immunofluorescence) *Bartonella henselae*, IgG antibody (indirect immunofluorescence)	Nonreactive <320
*NGS panel (genes tested)*	
*ASXL1, CEBPA, NPM1, ETV6, FLT3, CSF3R, DNMT3A, EZH2, GATA1, IDH1-2, IKZF1, RUNX1, SFEB1, TET2, WT1, TP53*	Unmutated
